# Reaching Out to Older Adults in Rural Communities by Utilizing Community Health Workers During a Pandemic

**DOI:** 10.1093/geroni/igab046.1226

**Published:** 2021-12-17

**Authors:** Jenny Knecht

**Affiliations:** Pennsylvania State University, University Park, Pennsylvania, United States

## Abstract

Older adults in rural communities have access, isolation, and technology barriers to healthcare that are exacerbated by the COVID- 19 pandemic. A shortage of healthcare professionals combined with limited resources and poor broadband access have limited their ability to benefit from telehealth. The pandemic has further worsened isolation in rural communities. This Age-Friendly Care, PA pilot study uses community health workers (CHW) as a bridge to connect isolated and underserved older adults with their healthcare team. The CHWs facilitate access to telehealth provided by a Federally Qualified Health Center (FQHC), and also provide “check-ins’ to housebound patients. The focus of the intervention is CHW delivered facilitation of telehealth and other supports to better manage their healthcare needs. We will describe the co-design of the project and discuss lessons learned in attempting to bridge the digital divide for rural older adults during and after the pandemic.

